# Species contributions to ecosystem process and function can be population dependent and modified by biotic and abiotic setting

**DOI:** 10.1098/rspb.2016.2805

**Published:** 2017-05-31

**Authors:** Daniel Wohlgemuth, Martin Solan, Jasmin A. Godbold

**Affiliations:** 1 Ocean and Earth Science, National Oceanography Centre Southampton, University of Southampton, Waterfront Campus, European Way, Southampton SO14 3ZH, UK; 2 Biological Sciences, University of Southampton, Highfield, Southampton, SO17 1BJ, UK

**Keywords:** bioirrigation, bioturbation, functional traits, functional diversity, population, trait variability

## Abstract

There is unequivocal evidence that altered biodiversity, through changes in the expression and distribution of functional traits, can have large impacts on ecosystem properties. However, trait-based summaries of how organisms affect ecosystem properties often assume that traits show constancy within and between populations and that species contributions to ecosystem functioning are not overly affected by the presence of other species or variations in abiotic conditions. Here, we evaluate the validity of these assumptions using an experiment in which three geographically distinct populations of intertidal sediment-dwelling invertebrates are reciprocally substituted. We find that the mediation of macronutrient generation by these species can vary between different populations and show that changes in biotic and/or abiotic conditions can further modify functionally important aspects of the behaviour of individuals within a population. Our results demonstrate the importance of knowing how, when, and why traits are expressed and suggest that these dimensions of species functionality are not sufficiently well-constrained to facilitate the accurate projection of the functional consequences of change. Information regarding the ecological role of key species and assumptions about the form of species–environment interactions needs urgent refinement.

## 1. Introduction

A wealth of empirical studies over the past two decades have provided unequivocal evidence that altering biodiversity leads to concomitant changes in ecosystem functioning that, ultimately, can affect the benefits that humans derive from ecological systems [1]. Indeed, recent consensus emphasizes the functional importance of individual species, rather than species diversity, in mediating ecosystem processes that are important in maintaining efficient and productive ecosystems [2–4]. This has revitalized interest in applying trait-based indices of functional diversity, in both terrestrial [5–7] and marine ecosystems [7–9], in order to provide a mechanistic understanding of the biotic control of ecosystem functioning and/or service delivery. While most of these approaches use non-phylogenetic biological attributes (i.e. physiological, morphological, or phenological characteristics [10]) to focus on how species mediate ecosystem functioning, they typically disregard variation in trait values (exceptions exist [11,12]) and, instead, focus on mean performance. In doing so, the contributory roles of species are assumed to show functional constancy in time and across space and, therefore, do not necessarily reflect the realized role of species [13]. Further, these perceptions are seldom challenged or interrogated and are infrequently explored empirically or objectively validated [14,15]. Nonetheless, these functional summaries are increasingly being adopted within predictive tools that incorporate community dynamics to project ecosystem responses to environmental change for the purposes of ecosystem management and planning [7,16,17].

As the allocation of species to a functional group and/or the assignment of functionally important traits is frequently based on single mean trait values per species [18,19], assessments of species contributions to functioning often underestimate the importance of intraspecific trait variation (but see [20]) and assume that an organism's functional effects and responses will be the same within and between populations [13,21]. However, the expression of functional traits within species is unlikely to be homogenously distributed, as individuals behave differently depending on the biotic and/or environmental conditions they experience [22–26]. Such context-dependent changes in trait expression, including, for example, responses to temperature [27], hydrodynamic regimes [28,29], resource availability and quality [30–32], or biotic interactions (e.g. predation [33,34]; competition [35]), can mean that the functional role of an individual may fundamentally change over time and across space, with corresponding transient effects on ecosystem properties [25,36,37].

Theory, as well as observations in plant communities [38], suggests that the relative importance of intraspecific variation in trait expression will decline with increasing scale as more variation is considered [39]. Here, we test this supposition in a marine system by exploring variability in sediment particle reworking activity, burrow ventilation behaviour, and the associated generation of nutrients for three distinct populations of three functionally contrasting sediment-dwelling invertebrate species that are common in mid-latitude eastern Atlantic and Mediterranean intertidal mudflats. Our *a priori* expectation was that undefined differences in location-specific environmental setting would lead to inter-population variation in behaviour that reflects differences in the extent and nature of organism–sediment coupling. A prominence of these sources of variation would emphasize the importance of the individual and/or population, rather than the species *per se*, and would highlight the need to incorporate sources of performance variability within biodiversity–ecosystem functioning models and ecosystem management strategies.

## 2. Methods

### (a) Experimental set-up and design

Surficial sediment (less than 3 cm depth, including surficial oxidized and subsurface reduced sediment) and fauna were collected in August 2014 from three sites from the northern (Ythan Estuary, 57°20′09.1″ N, 2°00′20.6″ W), central (Humber Estuary, 53°38′31.2″ N, 0°04′08.0″ E), and southern (Hamble Estuary, 50°52′23.1″ N, 1°18′49.3″ W) regions of the UK. We collected individuals of the gastropod *Hydrobia ulvae* and the mud shrimp *Corophium volutator* by sieving (>500 µm), and individuals of the polychaete *Hediste diversicolor* by hand. Sediment from each location was independently sieved (500 µm mesh) in a seawater bath to remove macrofauna, allowed to settle for 48 h (to retain the fine fraction, <63 µm) and thoroughly mixed. Sediment grain size parameters were measured using laser diffraction (Malvern Mastersizer 2000) and calculated using standard logarithmic graphical measures [40]. Total organic carbon (TOC) was determined by loss on ignition (electronic supplementary material, figure S1 and table S1).

**Figure 1.**
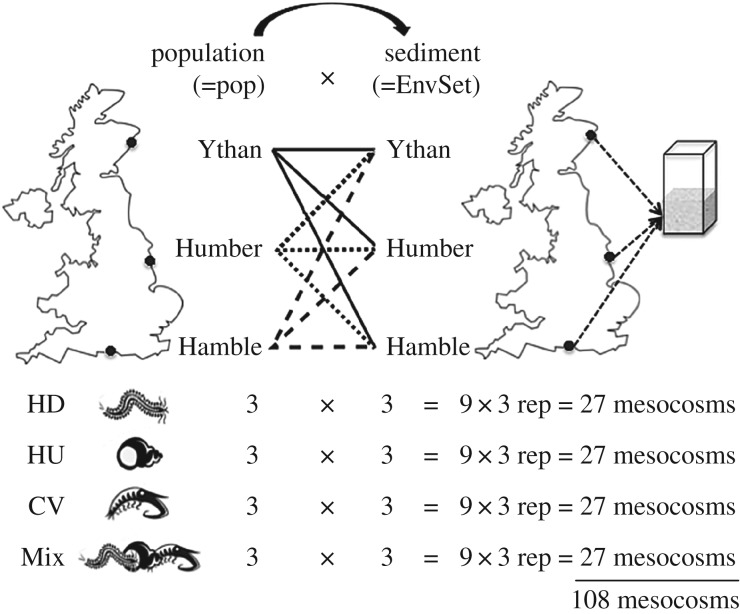
We adopted a full factorial experimental design consisting of three geographically distinct populations (Ythan, Humber, and Hamble estuaries) of invertebrate species (*H. ulvae*, HU; *C. volutator*, CV; *H. diversicolor*, HD) crossed with three environmental settings (sediment sourced from each geographical location). Species treatments included monocultures of each species (HD, HU, or CV) and a three-species mixture (Mix). Each treatment was replicated three times, giving a total of 108 aquaria. In addition, to distinguish the contribution of microbes and meiofauna from the activities of the macrofauna, we included additional aquaria that did not contain macrofauna (*n* = 9 environmental setting^−1^ = 27 aquaria) that were not included in statistical analyses.

Aquaria consisted of transparent square acrylic cores (internal dimensions, LWH, 12 × 12 × 35 cm), filled to approximately 10 cm with sediment overlain by approximately 20 cm of seawater (UV sterilized, 10 μm filtered, salinity 33) and maintained in a temperature-controlled water bath (14 ± 1°C, a value within the annual temperature range of all study site locations). After 24 h, the overlying water was exchanged to remove excess nutrients associated with assembly. We assembled replicate aquaria (*n* = 3) of each species in monoculture, and in a three species mixture (1 : 1 : 1), for each population (hereafter, Ythan, Humber, or Hamble). The species mixture allows determination of whether any observed variability that relates to environmental setting and/or population is conserved when biotic context is altered. To distinguish the effects of species interactions in the species mixture from the effects of density, we fixed biomass at 2 g wet weight aquarium^−1^ across all species treatment levels. To account for the effects of site-specific differences in environmental setting (mean ± s.d.) including differences in grain size distribution (Mz, sorting), organic carbon content (TC_org_) (Ythan, Mz = 49.4 ± 2 µm, sorting = 1.4 ± 0.08, TC_org_ = 9.3 ± 2.6%; Humber, Mz = 33.6 ± 1.1 µm, sorting = 1.9 ± 0.04, TC_org_ = 10.2 ± 2.2%; Hamble, Mz = 27.5 ± 0.9 µm, sorting = 2.4 ± 0.04, TC_org_ = 6.8 ± 0.1%; see electronic supplementary material, figure S1 and table S1) and any uncharacterized correlates, each species treatment was incubated in each sediment type. This allows us to distinguish the role of sediment conditions from that of species population effects (i.e. for each species treatment (4×): 3 populations × 3 environmental settings, in triplicate = 108 aquaria, figure 1). In addition, we included aquaria (*n* = 27) without macro-invertebrates to distinguish the contribution of macrofauna from that of the meiofauna and microbial processes. All aquaria were continually aerated and maintained under a 12 h light:dark regime for 12 days.

### (b) Quantification of ecosystem process and functioning

Faunal-mediated sediment particle reworking was estimated non-invasively using a sediment profile imaging camera (Canon 400D, set to 10 s exposure, aperture f5 and speed equivalent to ISO 400; 3 888 × 2 592 pixels, effective resolution = 63.1 µm pixel^−1^), modified to enable the preferential imaging of fluorescent-labelled particulate tracers (luminophores, pink colour, size class less than 125 µm; Brianclegg Ltd., UK) under UV light (f-SPI [41]). We analysed stitched composite images (RGB colour, JPEG compression, GMU Image Manipulation Program, v. 2.8.4, www.gimp.org/, Kimball S, Mattis P, GIMP (1995), date of access 01/10/2014), compiled from images of all four sides of each aquarium in a UV illuminated imaging box [42] after 12 days, using a custom-made semi-automated macro that runs within ImageJ (v. 1.47), a java-based public domain program developed at the US National Institutes of Health (http://rsb.info.nih.gov/ij/index.html, Rasband W, ImageJ (1997), date of access 01/10/2014). From these data, following [15], the mean (^f-SPI^L_mean,_) and maximum (^f-SPI^L_max_) depth of particle reworking was calculated. In addition, an estimate of surficial activity was determined using the maximum vertical deviation of the sediment–water interface (upper–lower limit; surface boundary roughness, SBR).

Burrow ventilation was estimated from absolute changes in the concentration of the inert tracer sodium bromide (Δ[Br^−^], mg l^−1^; negative values indicate increased activity) over a 4 h period during the daytime on day 12. Bromide concentrations were determined from pre-filtered (Fisherbrand, QL100, Ø 70 mm) water samples (5 ml, taken centrally, approximately 5 cm above the sediment–water interface) using a flow injection auto-analyser and standard protocols (FIAstar 5010 series, Foss-Tecator).

Nutrient concentrations ([NH_4_–N], [NO*_x_*–N], [PO_4_–P]) were quantified from pre-filtered (Fisherbrand, nylon 0.45 µm, Ø 25 mm) water samples (10 ml, taken centrally, approximately 5 cm above the sediment–water interface on day 12) using a flow injection auto-analyser (FIAstar 5010 series, Foss-Tecator) with an artificial seawater carrier solution.

### (c) Statistical analysis

For each species in monoculture (*H. diversicolor*, *H. ulvae*, *C. volutator*) and the three species mixture, we developed separate statistical models for each of the response variables (ecosystem processes: ^f-SPI^L_mean_, ^f-SPI^L_max_, SBR, Δ[Br^−^]; ecosystem functioning: [NH_4_–N], [NO*_x_*–N], [PO_4_–P]) with environmental setting and population as explanatory variables. As our main focus is to compare species contributions to functioning, and not to detect presence versus absence effects of species, aquaria that contained no invertebrates were not included in our statistical analyses but are presented for comparative purposes.

Initial linear models were assessed for normality (Q-Q-plot), heterogeneity of variance (plotted residual versus fitted values), and influential data points (cook's distance) [43]. When data exploration indicated variance heterogeneity, we applied generalized least squares (GLS) estimations that specifically incorporate variance in the residual spread with the explanatory variables, using appropriate variance functions (here *varIdent* for nominal explanatory variables) [43]. The optimal fixed structure was obtained by manual backward selection using the likelihood ratio test under maximum-likelihood (ML) estimation [43]. Coefficient tables are presented (electronic supplementary material, models S1–S23) without correction for the alpha-error, as Bonferroni correction increases the beta error and tends to obscure multiple significant results if *p*-values are moderate and the statistical power is low [44]. All statistical analyses were performed using the R statistical and programming environment [45] and the *nlme* package [46]. All data are provided in the electronic supplementary material, table S2.

## 3. Results

Our analyses confirm strong species-specific effects of environmental setting and/or population on ecosystem process and functioning across all of our response variables (for detail see electronic supplementary material, models S1–S23). Analysis of sediment properties confirm differences in bulk sediment descriptors (Mz, *σ*_l_, SK_l_, K_G_, particulate fraction < 63 µm, TOC; electronic supplementary material, figure S1 and table S1) between the three geographical locations. Overall, our results provide evidence that both differences in population and/or environmental setting can affect the way in which species moderate nutrient generation.

### (a) Effects on particle reworking and burrow ventilation

**Figure 2.**
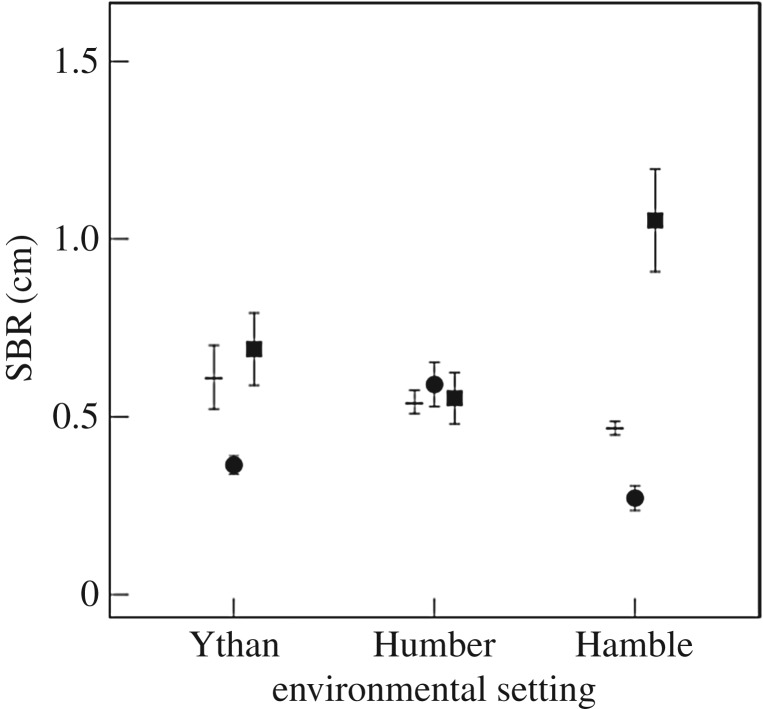
The effects of environmental setting on SBR (mean ± s.e., *n* = 3) for *H. ulvae* (circles) and *C. volutator* (squares). Observations without macrofauna (dash, *n* = 9) are shown for comparison.

SBR and the vertical redistribution of sediment particles (^f-SPI^L_mean_ and ^f-SPI^L_max_) are clearly influenced by a combination of interactive and additive effects of environmental setting and population that are dependent on species identity. We find that the faunal mediation of SBR is influenced by an independent effect of environmental setting (L-ratio = 14.33, d.f. = 2, *p* = <0.001) for *H. ulvae* (Humber > Ythan > Hamble, figure 2), or by a combination of the independent effects of environmental setting (Hamble > Ythan = Humber, L-ratio = 14.18, d.f. = 2, *p* < 0.001, figure 2) and population (Humber > Ythan = Hamble, L-ratio = 6.26, d.f. = 2, *p* = 0.044, electronic supplementary material, figure S2) for *C. volutator*. In contrast, we find no evidence that environmental setting or population affect the mediation of SBR when *H. diversicolor* is present in monoculture or when species are in mixture (both intercept only models; *F* = 1.44, d.f. = 2, *p* = 0.26 and *F* = 2.2, d.f. = 2, *p* = 0.13, respectively).

**Figure 3.**
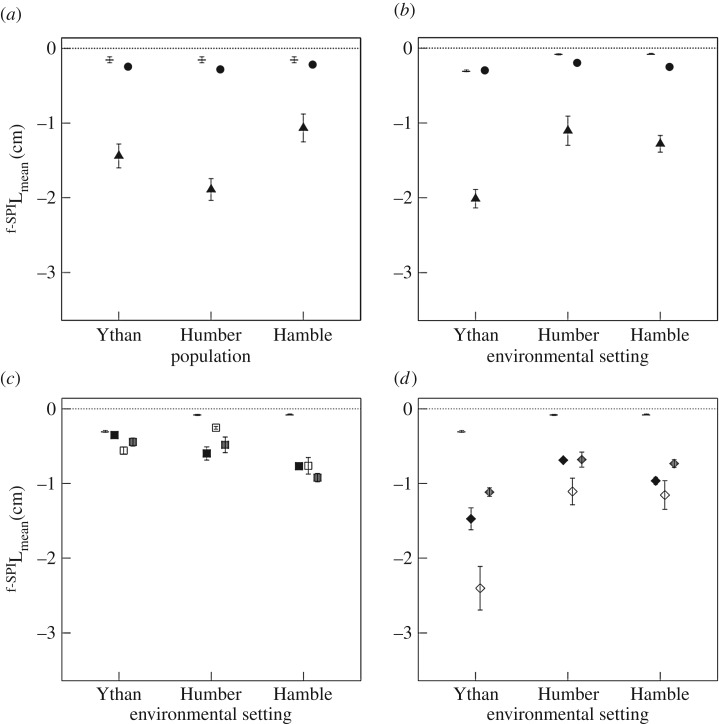
Independent effects of population (*a*) and environmental setting (*b*) on the mean depth of sediment particle reworking (^f-SPI^L_mean_, cm, mean ± s.e., *n* = 3) for *H. diversicolor* (triangles), *H. ulvae* (circles), and the interactive effect of environmental setting and population for (*c*) *C. volutator* (squares) and (*d*) the species mixture (diamonds). Observations without macrofauna (dash, *n* = 9) are shown for comparison. In panel (*c*) and (*d*), shadings indicate different populations: black, Ythan Estuary; white, Humber Estuary; grey, Hamble Estuary. The dotted line indicates the sediment surface and negative values indicate an increase in the net downward transport of sediment particles.

**Figure 4.**
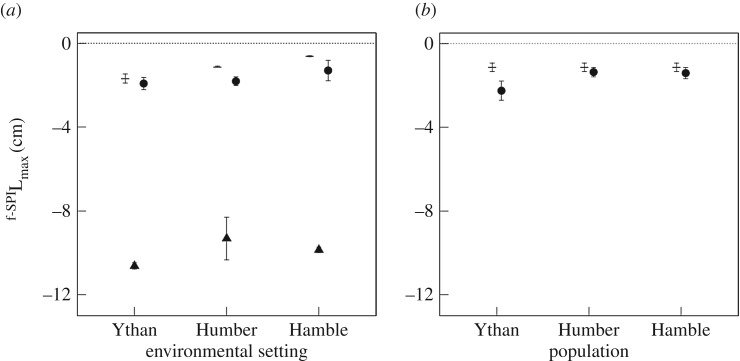
Effects of environmental setting (*a*) on the maximum depth of sediment particle reworking (^f-SPI^L_max_, cm, mean ± s.e., *n* = 3) for *H. diversicolor* (triangles) and *H. ulvae* (circles), and the effect of population (*b*) for *H. ulvae* (circles). Observations without macrofauna (dash, *n* = 9) are shown for comparison. The dotted line indicates the sediment surface and negative values indicate deeper net downward transport of sediment particles.

The mediation of ^f-SPI^L_mean_ (mean particle mixing depth, figure 3) in the presence of *H. diversicolor* and *H. ulvae* is influenced by the independent effects of environmental setting (*H. diversicolor*: *F* = 27.77, d.f. = 2, *p* < 0.0001; *H. ulvae, F* = 22.46, d.f. = 2, *p* < 0.0001) and population (*H. diversicolor*: *F* = 20.31, d.f. = 2, *p* < 0.0001; *H. ulvae, F* = 9.14, d.f. = 2, *p* < 0.001), but by the interactive effects of population × environmental setting in the presence of *C. volutator* (*C. volutator*: *F* = 4.72, d.f. = 4, *p* = 0.009; species mixture*,* L-ratio = 13.06, d.f. = 4, *p* = 0.01). In general, ^f-SPI^L_mean_ tends to be greatest for populations from the Humber (Humber > Ythan ≥ Hamble, figure 3*a*) and/or in sediments from the Ythan (Ythan > Hamble > Humber, figure 3*b*), although these patterns are not universal across all species treatments (figure 2). For ^f-SPI^L_max_ (figure 4), we find an effect of environmental setting for *H. diversicolor* (L-ratio = 11.89, d.f. = 2, *p* = 0.003), and independent effects of environmental setting and population (L-ratio = 31.74, d.f. = 2, *p* < 0.0001 and L-ratio = 8.35, d.f. = 2, *p* < 0.015, respectively) for *H. ulvae* (figure 4). ^f-SPI^L_max_ is deepest in sediment from the Ythan (figure 4*a*) and/or for the populations from the Ythan (figure 4*b*). There is also evidence for an interactive effect between environmental setting and population for the species mixture (L-ratio = 9.99, d.f. = 4, *p* = 0.041, electronic supplementary material, figure S3). In contrast, for *C. volutator*, we find no evidence that environmental setting or population are influential in determining ^f-SPI^L_max_ (intercept only model; *F* = 1.14, d.f. = 2, *p* = 0.34).

We find marginal effects of population on burrow ventilation ([ΔBr^−^]) for *H. diversicolor* and *C. volutator* (Ythan = Humber > Hamble: *F* = 3.43, d.f. = 2, *p* = 0.049 and Ythan > Humber = Hamble: *F* = 3.41, d.f. = 2, *p* = 0.05, respectively, electronic supplementary material, figure S4). There is no effect of environmental setting or population in the presence of *H. ulvae* (intercept only model; *F* = 2.34, d.f. = 2, *p* = 0.12) or when species are in mixture (intercept only model; *F* = 1.94, d.f. = 2, *p* = 0.17).

### (b) Effects on nutrient concentrations

**Figure 5.**
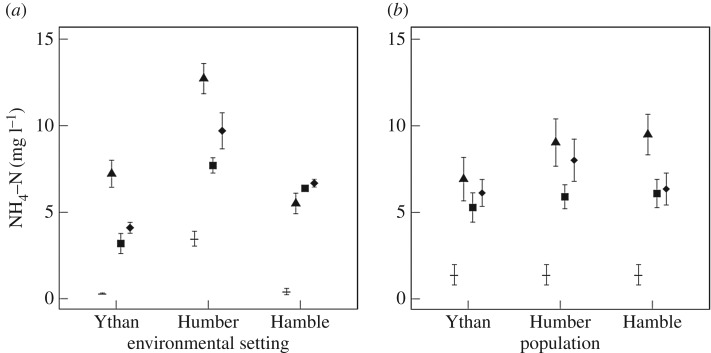
The effects of environmental setting (*a*) and population (*b*) on [NH_4_–N] (mg l^−1^, mean ± s.e., *n* = 3) for *H. diversicolor* (triangles), *C. volutator* (squares), and the species mixture (diamonds). Observations without macrofauna (dash, *n* = 9) are shown for comparison.

**Figure 6.**
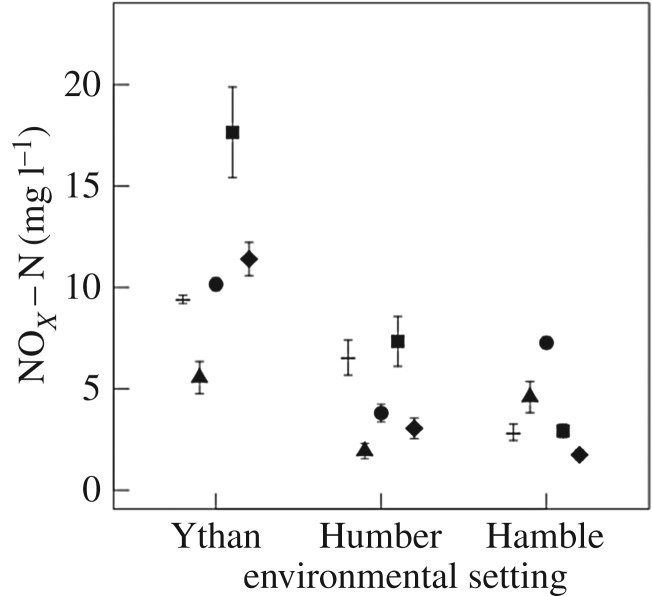
The effects of environmental setting on [NO*_X_*–N] (mg l^−1^, mean ± s.e., *n* = 3) for *H. diversicolor* (triangles), *H. ulvae* (circles), *C. volutator* (squares), and the species mixture (diamonds). Observations without macrofauna (dash, *n* = 9) are shown for comparison.

**Figure 7.**
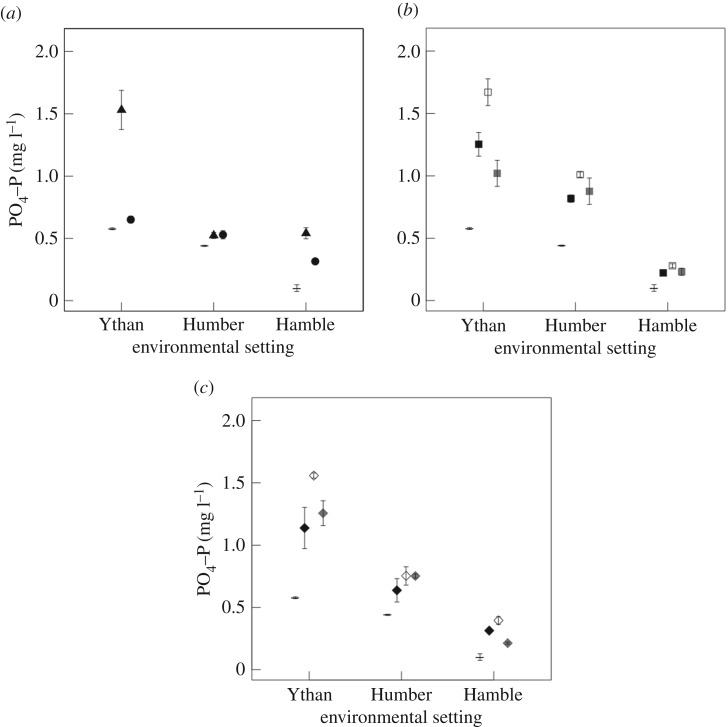
The effects of environmental setting on [PO_4_–P] (*a*, mg l^−1^, mean ± s.e., *n* = 3) for *H. diversicolor* (triangles) and *H. ulvae* (circles), and the interactive effect of environmental setting and population for *C. volutator* (*b*, squares) and the species mixture (c, diamonds). Observations without macrofauna (dash, *n* = 9) are shown for comparison. (*b*) and (*c*) shadings indicate different populations: black, Ythan Estuary; white, Humber Estuary; grey, Hamble Estuary.

Consistent effects of environmental setting are present across all species treatments, irrespective of nutrient identity, but the influence of population varies with nutrient identity ([NH_4_–N]: predominantly additive, figure 5; [NO*_x_*–N]: no effect, figure 6; [PO_4_–P]: no effect or interactive, figure 7). For [NH_4_–N] there are independent effects of both environmental setting and population for *H. diversicolor*, *C. volutator*, and the species mixture (environmental setting: *F* = 31.38, d.f. = 2, *p* < 0.0001; L-ratio = 37.25, d.f. = 2, *p* < 0.0001; L-ratio = 26.62, d.f. = 2, *p* < 0.0001, respectively; population: *F* = 4.16, d.f. = 2, *p* = 0.03; L-ratio = 16.84, d.f. = 2, *p* < 0.001; L-ratio = 9.6, d.f. = 2, *p* = 0.008, respectively). For *H. ulvae*, there is some weak evidence that these effects may be interactive (L-ratio = 9.55, d.f. = 4, *p* = 0.049, electronic supplementary material, figure S5). In general, [NH_4_–N] are higher in treatments with sediments from the Humber relative to those from the Hamble or the Ythan (figure 5*a*). The role of population is less pronounced, but populations of *H. diversicolor* and *C. volutator* from the Hamble and Humber return higher [NH_4_–N] relative to populations from the Ythan (figure 5*b*). For the species mixture, populations from the Humber return higher [NH_4_–N] than populations from the Hamble and Ythan (figure 5*b*).

We find a consistent effect of environmental setting on [NO*_x_*–N] across all of our species treatments (*H. diversicolor*: *F* = 7.79, d.f. = 2, *p* = 0.002; *H. ulvae*: *F* = 80.41, d.f. = 2, *p* < 0.0001; *C. volutator*: L-ratio = 25.04, d.f. = 2, *p* < 0.0001; species mixture: L-ratio = 52.94, d.f. = 2, *p* < 0.0001). For *H. diversicolor* and *H. ulvae* [NO*_x_*–N] are greater in sediments from the Hamble or Ythan (figure 6) relative to those of the Humber. In contrast, for *C. volutator* and the species mixture, the highest [NO*_x_*–N] are in sediments from the Ythan, followed by sediments from the Humber and Hamble (figure 6).

For [PO_4_–P] we find a single independent effect of environmental setting for *H. diversicolor* and *H. ulvae* (L-ratio = 21.65, d.f. = 2, *p* < 0.001; L-ratio = 54.01, d.f. = 2, *p* < 0.0001, respectively) and an interactive effect of environmental setting and population for *C. volutator* and the species mixture (L-ratio = 14.83, d.f. = 4, *p* = 0.005; L-ratio = 10.78, d.f. = 4, *p* = 0.029, respectively). [PO_4_–P] are higher in treatments containing sediments from the Ythan, followed by those with sediments from the Humber and Hamble (figure 7*a*). This trend is also reflected in the *C. volutator* and species mixture treatments, where the interaction is largely driven by population-specific differences within environmental settings (figure 7*b* and *c*).

## 4. Discussion

The use of functional traits to inform ecosystem management and policy relies on relating species functional effect traits, or functional diversity metrics, to ecosystem processes. However, concerns have been expressed about how important intraspecific variation is in defining functional trait structure [47–49] and how transferable functional designations may be across regions and with changing context, particularly in human-dominated landscapes [50,51]. Here, our experiments with intertidal sediment communities reveal that the presence of specific traits does not necessarily predetermine either the degree of species–environment interaction, or the way in which species mediate biogeochemical cycling; these can vary between populations and can be further moderated by dynamic shifts in abiotic and/or biotic circumstance [52]. Indeed, our findings indicate that the combined effects of abiotic/biotic conditions and historical precedent that are encapsulated in a specific location have the potential to determine the basal level of species–environmental interaction [53–55]. Individuals within a population may further regulate their own functional performance through additional morphological, physiological, or behavioural responses to transient changes in circumstance [25,31,34,36,56]. Hence, the net functional contributions of species to ecosystem properties will reflect the relative importance and interdependency of both short- and long-term processes that have altered, are altering, or are yet to fully alter the nature of species–environment coupling [26].

It is important to consider our findings in light of current practices that adopt single mean trait values to characterize how species mediate ecosystem properties [57]. Inherent in most functional metrics is the assumption that intraspecific trait variability is likely to be negligible relative to interspecific differences in species performance. Yet, with few exceptions [58], it is unlikely that functional effects will be synonymous with species taxonomy or be capable of being applied generically [14,59] because functional equivalence tends not to occur across local and regional scales, as well as across annual cycles [60]; a problem that will be compounded when multiple and/or more comprehensive trait descriptors are considered [15,61]. Although trait variation can be identified at local scales [62], scaling up will need to accommodate the long-term adjustment of species to local conditions and the history of environmental variation [63,64]. For example, one of our study species (*H. diversicolor*) is known to adapt its feeding strategy to local resource supply leading to morphological and behavioural differentiation [65] that, in turn, is likely to affect bioturbation activities of local populations. More widely, such adaptations can involve adjustments of morphological [65–67], behavioural [66–68], or physiological [69,70] traits in response to certain biotic and abiotic conditions. Indeed, as observed here, the functional role of species is not necessarily expressed to the same extent when species are in mixture, relative to when they are in monoculture. This is because the presence of interspecific interactions can positively or negatively affect the trait expression of individual species, altering *per capita* contributions to ecosystem functioning [71]. While the specific abiotic and/or biotic factors that lead to variation in trait expression are not easy to predict [22,72], the relationship between functional diversity and ecosystem properties has a strong theoretical base [73] and species responses to specific circumstances are well known. For example, the effects of timing [74,75] and environmental context [52] can moderate species–environment interactions and, albeit documented less frequently, the expression of functionally relevant traits [30,31] and/or behaviours [25,37,76]. Importantly, when the response of individuals to changing circumstances link to the effect traits that determine the functional contribution of an organism, the summed response of the assemblage can be sufficient to affect ecological patterns and processes at larger scales [16,77]. Conversely, when species–environment interactions decouple [78–80] or do not balance (abiotic > biotic control [81]), the underlying reciprocal relationship between species and the environment is minimized and the relative importance of biotic control may be diminished or masked [53].

While the intrinsic variability within species and the importance of local population adaptation have been recognized and are informing evolutionary thinking [82,83], equivalent information is yet to be fully incorporated into predictive models that explore the functional contribution of populations to ecosystem properties [49]. Our findings lend support to the growing consensus that community-level dynamics and intraspecific variability [13,39,84] need to be incorporated into ecological models when predicting the ecosystem consequences of altered biodiversity over large scales or extended time periods [7,16,17], especially when the risk of altered trait expression covaries with environmental forcing [85]. This means that more must be done to generate basic information on the hierarchical scaling of trait variance [86,87] and less reliance should be placed on macroecological and meta-analytical approaches that focus on point-based traits. Instead, a shift from species-based to individual-based ecology is necessary [13,84,87] and, as multiple trait information for individuals is not necessarily obtained by combining several trait databases, alternative statistical or modelling approaches that can fill data gaps and incorporate factors known to influence trait expression need to be developed [88]. When attempting to conserve the functional integrity of ecosystems under global change, a primary challenge for ecosystem management will be to account for the circumstances under which response and effect traits are linked [16], and when and where intraspecific versus interspecific trait variability are most influential [89].

## Supplementary Material

Sediment data, statistical model summary and additional figures

## Data accessibility

All data are provided in the electronic supplementary material, table S2.
