# Supplementary material for: Species contributions to ecosystem process and function can be population dependent and modified by biotic and abiotic setting
Source: Proc Biol Sci. 2017 May 31;284(1855):20162805. doi: 10.1098/rspb.2016.2805 (PMC5454255; doi:10.1098/rspb.2016.2805)
Supplement: Sediment data, statistical model summary and additional figures [file rspb20162805supp1.docx]

**Electronic supplementary material**

**Species contributions to ecosystem process and function can be population dependent and modified by biotic and abiotic setting**

Daniel Wohlgemuth^1^, Martin Solan^1^, Jasmin A. Godbold^1,2^

^1^Ocean and Earth Science, National Oceanography Centre Southampton, University of Southampton, Waterfront Campus, European Way, Southampton SO14 3ZH, UK,

^2^ Biological Sciences, University of Southampton, Highfield, Southampton, SO17 1BJ, UK.

Correspondence and requests for materials should be addressed to D.W. (email: [d.wohlgemuth@soton.ac.uk](mailto:d.wohlgemuth@soton.ac.uk))

**Sediment parameters**

Sediment parameters were measured by laser diffraction (Malvern Mastersizer 2000) at the Department of Geography, University of Cambridge, following standard protocols (available at: http://www.geog.cam.ac.uk/facilities/laboratories/techniques/). Particle size parameters were calculated using logarithmic graphical measures (Blott & Pye 2001).


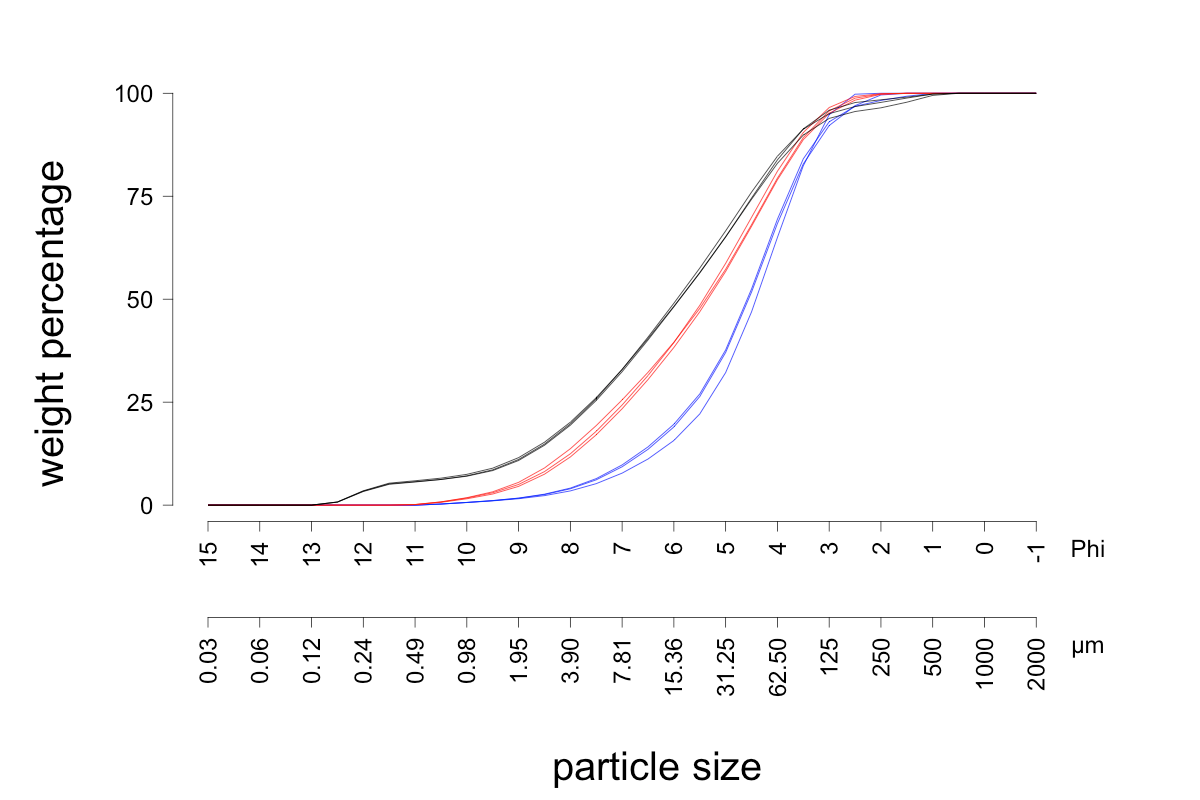


Figure S1: Cumulative sediment particle size distributions for the Ythan Estuary (blue), Humber Estuary (red) and Hamble Estuary (black).

Table S1: Sediment parameters (mean ± sd, n=3) for the three environmental settings used in the experiment.

| **Source of sediment** | **Mz**  **(µm)** | **Mz (Phi)** | **Sorting (µm)** | **Sorting (Phi)** | **Kurtosis (µm)** | **Kurtosis (Phi)** | **Skeweness (µm)** | **Skeweness (Phi)** | **Results below 63 µm (%)** | **TOC (%)** |
| --- | --- | --- | --- | --- | --- | --- | --- | --- | --- | --- |
| Ythan Estuary | 49.4  ± 2 | 4.7  ± 0.1 | 375.8  ± 21.4 | 1.4  ± 0.08 | 451.7  ± 3.6 | 1.1  ± 0.01 | 1208.8  ± 27.1 | -0.3  ± 0.03 | 68.8  ± 2.3 | 9.3  ± 2.6 |
| Humber Estuary | 33.6  ± 1.1 | 5.6  ± 0.1 | 274.3  ± 7.2 | 1.9  ± 0.04 | 540.9  ± 5.7 | 0.9  ± 0.02 | 1151.4  ± 12.2 | -0.2  ± 0.02 | 80.1  ± 1.1 | 10.2  ± 2.2 |
| Hamble Estuary | 27.5  ± 0.9 | 6.1  ± 0.04 | 189.1  ± 4.6 | 2.4  ± 0.04 | 449.7  ± 9.55 | 1.2  ± 0.03 | 1167.6  20.4 | -0.2  ± 0.03 | 84.0  ± 0.9 | 6.8  ± 0.1 |

**Statistical model summary**

Summary of the statistical models analysing each species and the three species mixture separately (Model S1 to S23). For each model we list the initial linear regression model and the minimal adequate model. When variance homogeneity was violated we used a linear regression with generalized least squares (GLS) estimation. We present a summary of the coefficient tables for single terms. The coefficients indicate the relative performance of each treatment level in relation to the re-levelled baseline (as indicated). Coefficients ± SE, t-values and respective significance values are presented.

**Abbreviations:**

**(i) Explanatory variables**

EnvSet, environmental setting

Pop, population

**(ii) Response variables**

SBR, surface boundary roughness (cm)

^f-SPI^L_mean,_ , mean mixed depth of particle reworking (cm)

^f-SPI^L_max,_ , maximum mixed depth of particle reworking (cm)

Δ[Br^-^], burrow ventilation (mg L^-1^)

[NH_4_-N], NH_4_-N concentration (mg L^-1^)

[NO_x_-N], NO_2_-N + NO_3_-N concentration (mg L^-1^)

[PO_4_-P], PO_4_-P concentration (mg L^-1^)

**Data:** All data used in the analyses are provided as Table S2.

**Model S1**: Surface boundary roughness (SBR, cm) - *Hydrobia ulvae*

Initial linear regression model:

lm(SBR ~ EnvSet+Pop+EnvSet:Pop)

Minimal adequate model:

gls(SBR ~ EnvSet, weights = varIdent(form = ~1|EnvSet), method = ‘REML’)

Intercept ± SE (when baseline is for Ythan Estuary): 0.364 ± 0.026, t = 14.010, p < 0.0001

Coefficient table for EnvSet

|  | Ythan | Humber | Hamble |
| --- | --- | --- | --- |
| Ythan | / | -0.226 ± 0.067  -3.361  **0.003** | 0.093 ± 0.043  2.149  **0.042** |
| Humber | 0.226 ± 0.067  3.361  **0.003** | / | 0.319 ± 0.071  4.491  **<0.001** |
| Hamble | -0.093 ± 0.043  -2.149  **0.042** | -0.319 ± 0.071  -4.491  **<0.001** | / |

**Model S2:** Surface boundary roughness (SBR, cm) - *Corophium volutator*

Initial linear regression model:

lm(SBR ~ EnvSet+Pop+EnvSet:Pop)

Minimal adequate model:

gls(SBR ~ EnvSet+Pop, weights = varIdent(form = ~1|Pop), method = ‘REML’)

Intercept ± SE (when baseline is for Ythan Estuary for EnvSet and Pop): 0.552 ± 0.082, t = 6.764, p < 0.0001

Coefficient table for EnvSet

|  | Ythan | Humber | Hamble |
| --- | --- | --- | --- |
| Ythan | / | 0.120 ± 0.100  1.198  0.244 | -0.305 ± 0.100  -3.040  **0.006** |
| Humber | -0.120 ± 0.100  -1.198  0.244 | / | -0.425 ± 0.100  -4.239  **<0.001** |
| Hamble | 0.305 ± 0.100  3.040  **0.006** | 0.425 ± 0.100  4.239  **<0.001** | / |

Coefficient table for Pop

|  | Ythan | Humber | Hamble |
| --- | --- | --- | --- |
| Ythan | / | -0.399 ± 0.149  -2.679  **0.014** | -0.056 ± 0.086  -0.653  0.521 |
| Humber | 0.399 ± 0.149  2.679  **0.014** | / | 0.343 ± 0.152  2.260  **0.034** |
| Hamble | 0.056 ± 0.086  0.653  0.521 | -0.343 ± 0.152  -2.260  **0.034** | / |

**Model S3**: Mean mixed depth of particle reworking (^f-SPI^L_mean_, cm) - *Hediste diversicolor*

Initial linear regression model:

lm(^f-SPI^L_mean_ ~ EnvSet+Pop+EnvSet:Pop)

Minimal adequate model:

lm(^f-SPI^L_mean_ ~ EnvSet+Pop)

Intercept ± SE (when baseline is for Ythan Estuary for EnvSet and Pop): 1.987 ± 0.119, t = 16.818, p < 0.0001

Coefficient table for EnvSet

|  | Ythan | Humber | Hamble |
| --- | --- | --- | --- |
| Ythan | / | 0.909 ± 0.129  7.025  **<0.0001** | 0.734 ± 0.129  5.668  **<0.0001** |
| Humber | -0.909 ± 0.129  -7.025  **<0.0001** | / | -0.176 ± 0.129  -1.356  0.189 |
| Hamble | -0.734 ± 0.129  -5.668  **<0.0001** | 0.176 ± 0.129  1.356  0.189 | / |

Coefficient table for Pop

|  | Ythan | Humber | Hamble |
| --- | --- | --- | --- |
| Ythan | **/** | -0.450 ± 0.129  -3.474  **0.002** | 0.374 ± 0.129  2.891  **0.008** |
| Humber | 0.450 ± 0.129  3.474  **0.002** | / | 0.824 ± 0.129  6.364  **<0.0001** |
| Hamble | -0.374 ± 0.129  -2.891  **0.008** | -0.824 ± 0.129  -6.364  **<0.0001** | / |

**Model S4**: Mean mixed depth of particle reworking (^f-SPI^L_mean_, cm) - *Hydrobia ulvae*

Initial linear regression model:

lm(^f-SPI^L_mean_ ~ EnvSet+Pop+EnvSet:Pop)

Minimal adequate model:

lm(^f-SPI^L_mean_ ~ EnvSet+Pop)

Intercept ± SE (when baseline is for Ythan Estuary for EnvSet and Pop): 0.294 ± 0.014, t = 21.541, p < 0.0001

Coefficient table for EnvSet

|  | Ythan | Humber | Hamble |
| --- | --- | --- | --- |
| Ythan | / | 0.100 ± 0.150  6.694  **<0.0001** | 0.046 ± 1.150  3.054  **0.006** |
| Humber | -0.100 ± 0.150  -6.694  **<0.0001** | / | -0.054 ± 0.150  -3.640  **0.001** |
| Hamble | -0.046 ± 1.150  -3.054  **0.006** | 0.054 ± 0.150  3.640  **0.001** | / |

Coefficient table for Pop

|  | Ythan | Humber | Hamble |
| --- | --- | --- | --- |
| Ythan | **/** | -0.036 ± 0.150  -2.411  **0.025** | 0.028 ± 0.150  1.852  0.078 |
| Humber | 0.036 ± 0.150  2.411  **0.025** | / | 0.064 ± 0.150  4.263  **0.0003** |
| Hamble | -0.028 ± 0.150  -1.852  0.078 | -0.064 ± 0.150  -4.263  **0.0003** | / |

**Model S5**: Mean mixed depth of particle reworking (^f-SPI^L_mean_, cm) - *Corophium volutator*

Initial linear regression model:

lm(^f-SPI^L_mean_ ~ EnvSet+Pop+EnvSet:Pop)

Minimal adequate model:

lm(^f-SPI^L_mean_ ~ EnvSet+Pop+EnvSet:Pop)

**Model S6**: Mean mixed depth of particle reworking (^f-SPI^L_mean_, cm) - species mixture

Initial linear regression model:

lm(^f-SPI^L_mean_ ~ EnvSet+Pop+EnvSet:Pop)

Minimal adequate model:

gls(^f-SPI^L_mean_ ~ EnvSet+Pop+EnvSet:Pop, weights = varIdent(form = ~1|Pop), method = ‘REML’))

**Model S7**: Maximum mixed depth of particle reworking (^f-SPI^L_max_, cm) - *Hediste diversicolor*

Initial linear regression model:

lm(^f-SPI^L_max_ ~ EnvSet+Pop+EnvSet:Pop)

Minimal adequate model:

gls(^f-SPI^L_max_ ~ EnvSet, weights = varIdent(form = ~1|EnvSet), method = ‘REML’)

Intercept ± SE (when baseline is for Ythan Estuary): 10.627 ± 0.151, t = 70.244, p < 0.0001

Coefficient table for EnvSet

|  | Ythan | Humber | Hamble |
| --- | --- | --- | --- |
| Ythan | / | 1.313 ± 1.028  1.277  0.214 | 0.771 ± 0.205  3.768  **0.001** |
| Humber | -1.313 ± 1.028  -1.277  0.214 | / | -0.542 ± 1.026  -0.529  0.602 |
| Hamble | -0.771 ± 0.205  -3.768  **0.001** | 0.542 ± 1.026  0.529  0.602 | / |

**Model S8**: Maximum mixed depth of particle reworking (^f-SPI^L_max_, cm) - *Hydrobia ulvae*

Initial linear regression model:

lm(^f-SPI^L_max_ ~ EnvSet+Pop+EnvSet:Pop)

Minimal adequate model:

gls(^f-SPI^L_max_ ~ EnvSet+Pop, weights = varIdent(form = ~1|EnvSet×Pop), method = ‘REML’)

Intercept ± SE (when baseline is for Ythan Estuary for EnvSet and Pop): 2.245 ± 0.345, t = 6.516, p < 0.0001

Coefficient table for EnvSet

|  | Ythan | Humber | Hamble |
| --- | --- | --- | --- |
| Ythan | / | 0.026 ± 0.220  0.118  0.907 | 0.873 ± 0.181  4.815  **0.0001** |
| Humber | -0.026 ± 0.220  -0.118  0.907 | / | 0.847 ± 0.134  6.304  **<0.0001** |
| Hamble | -0.873 ± 0.181  -4.815  **0.0001** | -0.847 ± 0.134  -6.304  **<0.0001** | / |

Coefficient table for Pop

|  | Ythan | Humber | Hamble |
| --- | --- | --- | --- |
| Ythan | / | 0.632 ± 0.317  1.992  0.059 | 0.755 ± 0.315  2.399  **0.025** |
| Humber | -0.632 ± 0.317  -1.992  0.059 | / | 0.123 ± 0.058  2.119  **0.046** |
| Hamble | -0.755 ± 0.315  -2.399  **0.025** | -0.123 ± 0.058  -2.119  **0.046** | / |

**Model S9**: Maximum mixed depth of particle reworking (^f-SPI^L_max_, cm) - species mixture

Initial linear regression model:

lm(^f-SPI^L_max_ ~ EnvSet+Pop+EnvSet:Pop)

Minimal adequate model:

gls(^f-SPI^L_max_ ~ EnvSet+Pop+EnvSet:Pop, weights = varIdent(form = ~1|EnvSet), method = ‘REML’)

**Model S10**: Burrow ventilation (Δ[Br^-^], mg L^-1^) - *Hediste diversicolor*

Initial linear regression model:

lm(Δ[Br^-^] ~ EnvSet+Pop+EnvSet:Pop)

Minimal adequate model:

lm(Δ[Br^-^] ~ Pop)

Intercept ± SE (when baseline is for Ythan Estuary for Pop): -69.213 ± 10.654, t = -6.496, p < 0.0001

Coefficient table for Pop

|  | Ythan | Humber | Hamble |
| --- | --- | --- | --- |
| Ythan | / | -9.302 ± 15.067  -0.617  0.543 | -37.849 ± 15.067  -2.512  **0.019** |
| Humber | 9.302 ± 15.067  0.617  0.543 | / | -28.548 ± 15.067  -1.895  0.070 |
| Hamble | 37.849 ± 15.067  2.512  **0.019** | 28.548 ± 15.067  1.895  0.070 | / |

**Model S11**: Burrow ventilation (Δ[Br^-^], mg L^-1^) - *Corophium volutator*

Initial linear regression model:

lm(Δ[Br^-^] ~ EnvSet+Pop+EnvSet:Pop)

Minimal adequate model:

lm(Δ[Br^-^] ~ Pop)

Intercept ± SE (when baseline is for Ythan Estuary for Pop): -45.270 ± 10.520, t = -4.303, p = 0.0002

Coefficient table for Pop

|  | Ythan | Humber | Hamble |
| --- | --- | --- | --- |
| Ythan | / | -37.600 ± 14.881  -2.527  **0.019** | -27.270 ± 14.881  -1.833  0.079 |
| Humber | 37.600 ± 14.881  2.527  **0.019** | / | 10.325 ± 14.881  0.694  0.494 |
| Hamble | 27.270 ± 14.881  1.833  0.079 | -10.325 ± 14.881  -0.694  0.494 | / |

**Model S12**: NH_4_-N concentration ([NH_4_-N], mg L^-1^) - *Hediste diversicolor*

Initial linear regression model:

lm([NH_4_-N] ~ EnvSet+Pop+EnvSet:Pop)

Minimal adequate model:

lm([NH_4_-N] ~ EnvSet+Pop)

Intercept ± SE (when baseline is for Ythan Estuary for EnvSet and Pop): 5.658 ± 0.869, t = 6.508, p < 0.0001

Coefficient table for EnvSet

|  | Ythan | Humber | Hamble |
| --- | --- | --- | --- |
| Ythan | / | -5.502 ± 0.952  -5.778  **<0.0001** | 1.720 ± 0.952  1.805  0.085 |
| Humber | 5.502 ± 0.952  5.778  **<0.0001** | / | 7.222 ± 0.952  7.582  **<0.0001** |
| Hamble | -1.720 ± 0.952  -1.805  0.085 | -7.222 ± 0.952  -7.582  **<0.0001** | / |

Coefficient table for Pop

|  | Ythan | Humber | Hamble |
| --- | --- | --- | --- |
| Ythan | / | -2.114 ± 0.952  -2.220  **0.037** | -2.577 ± 0.952  -2.706  **0.013** |
| Humber | 2.114 ± 0.952  2.220  **0.037** | / | -0.463 ± 0.952  -0.486  0.632 |
| Hamble | 2.577 ± 0.952  2.706  **0.013** | 0.463 ± 0.952  0.486  0.632 | / |

**Model S13**: NH_4_-N concentration ([NH_4_-N], mg L^-1^) - *Hydrobia ulvae*

Initial linear regression model:

lm([NH_4_-N] ~ EnvSet+Pop+EnvSet:Pop)

Minimal adequate model:

gls([NH_4_-N] ~ EnvSet+Pop+EnvSet:Pop, weights = varIdent(form = ~1|EnvSet), method = ‘REML’)

**Model S14**: NH_4_-N concentration ([NH_4_-N], mg L^-1^) - *Corophium volutator*

Initial linear regression model:

lm([NH_4_-N] ~ EnvSet+Pop+EnvSet:Pop)

Minimal adequate model:

gls([NH_4_-N] ~ EnvSet+Pop, weights = varIdent(form = ~1| EnvSet×Pop), method = ‘REML’)

Intercept ± SE (when baseline is for Ythan Estuary for EnvSet and Pop): 2.414 ± 0.141, t = 17.100, p < 0.0001

Coefficient table for EnvSet

|  | Ythan | Humber | Hamble |
| --- | --- | --- | --- |
| Ythan | / | -4.442 ± 0.235  -18.886  **<0.0001** | -3.483 ± 0.148  -23.483  **<0.0001** |
| Humber | 4.442 ± 0.235  18.886  **<0.0001** | / | 0.960 ± 0.184  5.223  **<0.0001** |
| Hamble | 3.483 ± 0.148  23.483  **<0.0001** | -0.960 ± 0.184  -5.223  **<0.0001** | / |

Coefficient table for Pop

|  | Ythan | Humber | Hamble |
| --- | --- | --- | --- |
| Ythan | / | -0.801 ± 0.073  -11.026  **<0.0001** | -0.699 ± 0.215  -3.246  **0.004** |
| Humber | 0.801 ± 0.073  11.026  **<0.0001** | / | 0.103 ± 0.216  0.475  0.640 |
| Hamble | 0.699 ± 0.215  3.246  **0.004** | -0.103 ± 0.216  -0.475  0.640 | / |

**Model S15**: NH_4_-N concentration ([NH_4_-N], mg L^-1^) - species mixture

Initial linear regression model:

lm([NH_4_-N] ~ EnvSet+Pop+EnvSet:Pop)

Minimal adequate model:

gls([NH_4_-N] ~ EnvSet+Pop, weights = varIdent(form = ~1|EnvSet), method = ‘REML’)

Intercept ± SE (when baseline is for Ythan Estuary for EnvSet and Pop): 3.950 ± 0.385, t = 10.271, p < 0.0001

Coefficient table for EnvSet

|  | Ythan | Humber | Hamble |
| --- | --- | --- | --- |
| Ythan | / | -5.604 ± 1.020  -5.493  **<0.0001** | -2.574 ± 0.366  -7.041  **<0.0001** |
| Humber | 5.604 ± 1.020  5.493  **<0.0001** | / | 3.030 ± 0.976  3.104  **0.005** |
| Hamble | 2.574 ± 0.366  7.041  **<0.0001** | -3.030 ± 0.976  -3.104  **0.005** | / |

Coefficient table for Pop

|  | Ythan | Humber | Hamble |
| --- | --- | --- | --- |
| Ythan | / | -0.771 ± 0.333  -2.317  **0.030** | 0.324 ± 0.333  0.973  0.341 |
| Humber | 0.771 ± 0.333  2.317  **0.030** | / | 1.095 ± 0.333  3.290  **0.003** |
| Hamble | -0.324 ± 0.333  -0.973  0.341 | -1.095 ± 0.333  -3.290  **0.003** | / |

**Model S16**: NO_X_-N concentration ([NO_X_-N], mg L^-1^) - *Hediste diversicolor*

Initial linear regression model:

lm([NO_X_-N] ~ EnvSet+Pop+EnvSet:Pop)

Minimal adequate model:

lm([NO_X_-N] ~ EnvSet)

Intercept ± SE (when baseline is for Ythan Estuary): 5.555 ± 0.672, t = 8.270, p < 0.0001

Coefficient table for EnvSet

|  | Ythan | Humber | Hamble |
| --- | --- | --- | --- |
| Ythan | / | 3.620 ± 0.95  3.810  **<0.001** | 0.961 ± 0.95  1.012  0.322 |
| Humber | -3.620 ± 0.95  -3.810  **<0.001** | / | -2.659 ± 0.95  -2.799  **0.01** |
| Hamble | -0.961 ± 0.95  -1.011  0.322 | 2.659 ± 0.95  2.799  **0.01** | / |

**Model S17**: NO_X_-N concentration ([NO_X_-N], mg L^-1^) - *Hydrobia ulvae*

Initial linear regression model:

lm([NO_X_-N] ~ EnvSet+Pop+EnvSet:Pop)

Minimal adequate model:

lm([NO_X_-N] ~ EnvSet)

Intercept ± SE (when baseline is for Ythan Estuary): 10.15 ± 0.355, t = 28.54, p < 0.0001

Coefficient table for EnvSet

|  | Ythan | Humber | Hamble |
| --- | --- | --- | --- |
| Ythan | / | 6.35 ± 0.502  12.660  **<0.0001** | 2.884 ± 0.502  5.750  **<0.0001** |
| Humber | -6.35 ± 0.502  -12.660  **<0.0001** | / | -3.466 ± 0.502  -6.914  **<0.0001** |
| Hamble | -2.884 ± 0.502  -5.750  **<0.0001** | 3.466 ± 0.502  6.914  **<0.0001** | / |

**Model S18**: NO_X_-N concentration ([NO_X_-N], mg L^-1^) - *Corophium volutator*

Initial linear regression model:

lm([NO_X_-N] ~ EnvSet+Pop+EnvSet:Pop)

Minimal adequate model:

gls([NO_X_-N] ~ EnvSet, weights = varIdent(form = ~1| EnvSet×Pop), method = ‘REML’)

Intercept ± SE (when baseline is for Ythan Estuary): 17.648 ± 2.235, t = 7.896, p < 0.0001

Coefficient table for EnvSet

|  | Ythan | Humber | Hamble |
| --- | --- | --- | --- |
| Ythan | / | 10.309 ± 2.551  4.042  **<0.001** | 14.722 ± 2.609  6.512  **<0.0001** |
| Humber | -10.309 ± 2.551  -4.042  **<0.001** | / | 4.413 ± 1.276  3.460  **0.002** |
| Hamble | -14.722 ± 2.609  -6.512  **<0.0001** | -4.413 ± 1.276  -3.460  **0.002** | / |

**Model S19**: NO_X_-N concentration ([NO_X_-N], mg L^-1^) - species mixture

Initial linear regression model:

lm([NO_X_-N] ~ EnvSet+Pop+EnvSet:Pop)

Minimal adequate model:

gls([NO_X_-N] ~ EnvSet, weights = varIdent(form = ~1|EnvSet×Pop), method = ‘REML’))

Intercept ± SE (when baseline is for Ythan Estuary): 12.349 ± 0.102, t = 121.518, p < 0.0001

Coefficient table for EnvSet

|  | Ythan | Humber | Hamble |
| --- | --- | --- | --- |
| Ythan | / | 9.546 ± 0.434  21.980  **<0.0001** | 10.702 ± 0.236  45.379  **<0.0001** |
| Humber | -9.546 ± 0.434  -21.980  **<0.0001** | / | 1.156 ± 0.473  2.444  **0.022** |
| Hamble | -10.702 ± 0.236  -45.379  **<0.0001** | -1.156 ± 0.473  -2.444  **0.022** | / |

**Model S20**: PO_4_-P concentration ([PO_4_-P], mg L^-1^) - *Hediste diversicolor*

Initial linear regression model:

lm([PO_4_-P] ~ EnvSet+Pop+EnvSet:Pop)

Minimal adequate model:

gls([PO_4_-P] ~ EnvSet, weights = varIdent(form = ~1|EnvSet), method = ‘REML’)

Intercept ± SE (when baseline is for Ythan Estuary): 1.530 ± 0.157, t = 9.741, p < 0.0001

Coefficient table for EnvSet

|  | Ythan | Humber | Hamble |
| --- | --- | --- | --- |
| Ythan | / | 1.008 ± 0.159  6.337  **<0.0001** | 0.990 ± 0.163  6.064  **<0.0001** |
| Humber | -1.008 ± 0.159  -6.337  **<0.0001** | / | -0.018 ± 0.051  -0.356  0.725 |
| Hamble | -0.990 ± 0.163  -6.064  **<0.0001** | 0.018 ± 0.051  0.356  0.725 | / |

**Model S21**: PO_4_-P concentration ([PO_4_-P], mg L^-1^) - *Hydrobia ulvae*

Initial linear regression model:

lm([PO_4_-P] ~ EnvSet+Pop+EnvSet:Pop)

Minimal adequate model:

gls([PO_4_-P] ~ EnvSet, weights = varIdent(form = ~1|Pop), method = ‘REML’)

Intercept ± SE (when baseline is for Ythan Estuary): 0.620 ± 0.013, t = 46.206, p < 0.0001

Coefficient table for EnvSet

|  | Ythan | Humber | Hamble |
| --- | --- | --- | --- |
| Ythan | / | 0.119 ± 0.019  6.277  **<0.0001** | 0.300 ± 0.019  15.778  **<0.0001** |
| Humber | -0.119 ± 0.019  -6.277  **<0.0001** | / | 0.180 ± 0.019  9.501  **<0.0001** |
| Hamble | -0.300 ± 0.019  -15.778  **<0.0001** | -0.180 ± 0.019  -9.501  **<0.0001** | / |

**Model S22**: PO_4_-P concentration ([PO_4_-P], mg L^-1^) - *Corophium volutator*

Initial linear regression model:

lm([PO_4_-P] ~ EnvSet+Pop+EnvSet:Pop)

Minimal adequate model:

gls([PO_4_-P] ~ EnvSet+Pop+EnvSet:Pop, weights = varIdent(form = ~1|EnvSet), method = ‘REML’)

**Model S23**: PO_4_-P concentration ([PO_4_-P], mg L^-1^) - species mixture

Initial linear regression model:

lm([PO_4_-P] ~ EnvSet+Pop+EnvSet:Pop)

Minimal adequate model:

gls([PO_4_-P] ~ EnvSet+Pop+EnvSet:Pop, weights = varIdent(form = ~1|EnvSet), method = ‘REML’)

Table S2: Summary of data used for statistical analysis. Data in the absence of macrofauna is shown for comparison but was not included in the analyses. EnvSet = environmental setting, Pop = population, SID = species identity (HD = *Hediste diversicolor*, HU = *Hydrobia ulvae*, CV = *Corophium volutator,* Mix = species mixture, cntrl = no macrofauna), Ha = Hamble Estuary, Hu = Humber Estuary, Y = Ythan Estuary

| Env  Set | Pop | SID | Repli-cate | ^f-SPI^L_mean_ (cm) | ^f-SPI^L_max_ (cm) | SBR  (cm) | Δ[Br^-^] (mg L^-1^) | [NH_4_-N] (mg L^-1^) | [NO_X_-N] (mg L^-1^) | [PO_4_-P] (mg L^-1^) |
| --- | --- | --- | --- | --- | --- | --- | --- | --- | --- | --- |
| Ha | Ha | HD | 1 | 0.915 | 10.465 | 0.850 | -20.160 | 6.552 | 3.124 | 0.358 |
| Ha | Ha | HD | 2 | 1.126 | 10.438 | 0.514 | -24.325 | 7.743 | 2.884 | 0.683 |
| Ha | Ha | HD | 3 | 0.965 | 9.608 | 0.746 | -36.740 | 5.745 | 4.306 | 0.596 |
| Ha | Ha | HU | 1 | 0.236 | 0.605 | 0.384 | 1.218 | 1.096 | 7.115 | 0.322 |
| Ha | Ha | HU | 2 | 0.208 | 0.575 | 0.316 | -11.459 | 1.384 | 6.298 | 0.329 |
| Ha | Ha | HU | 3 | 0.230 | 0.668 | 0.063 | -5.637 | 0.655 | 6.214 | 0.209 |
| Ha | Ha | CV | 1 | 0.928 | 2.839 | 0.969 | 7.622 | 6.697 | 3.676 | 0.268 |
| Ha | Ha | CV | 2 | 1.018 | 2.606 | 0.917 | -1.636 | 6.089 | 2.899 | 0.248 |
| Ha | Ha | CV | 3 | 0.821 | 2.380 | 0.467 | -8.667 | 6.817 | 1.529 | 0.175 |
| Ha | Ha | Mix | 1 | 0.638 | 4.650 | 0.422 | -70.389 | 6.499 | 0.882 | 0.203 |
| Ha | Ha | Mix | 2 | 0.744 | 8.680 | 1.237 | -7.093 | 6.007 | 1.393 | 0.230 |
| Ha | Ha | Mix | 3 | 0.819 | 7.751 | 0.776 | -34.825 | 6.096 | 1.788 | 0.204 |
| Ha | Hu | HD | 1 | 1.667 | 9.746 | 0.831 | -76.752 | 4.782 | 5.315 | 0.657 |
| Ha | Hu | HD | 2 | 1.868 | 9.639 | 0.638 | 15.830 | 7.952 | 0.774 | 0.731 |
| Ha | Hu | HD | 3 | 1.454 | 9.392 | 0.775 | -62.558 | 4.637 | 5.226 | 0.456 |
| Ha | Hu | HU | 1 | 0.279 | 0.780 | 0.302 | -22.948 | 0.765 | 6.239 | 0.293 |
| Ha | Hu | HU | 2 | 0.291 | 0.807 | 0.271 | -44.985 | 0.491 | 7.694 | 0.332 |
| Ha | Hu | HU | 3 | 0.242 | 0.638 | 0.139 | 33.476 | 0.769 | 7.364 | 0.345 |
| Ha | Hu | CV | 1 | 0.550 | 2.731 | 1.010 | -12.575 | 6.604 | 2.191 | 0.260 |
| Ha | Hu | CV | 2 | 0.927 | 3.941 | 1.280 | 20.377 | 6.797 | 4.248 | 0.310 |
| Ha | Hu | CV | 3 | 0.815 | 3.443 | 2.052 | -18.774 | 6.704 | 4.517 | 0.265 |
| Ha | Hu | Mix | 1 | 1.521 | 10.379 | 1.459 | -33.554 | 6.807 | 3.122 | 0.337 |
| Ha | Hu | Mix | 2 | 0.872 | 8.576 | 0.998 | 46.851 | 7.767 | 0.684 | 0.406 |
| Ha | Hu | Mix | 3 | 1.072 | 9.474 | 0.864 | -11.443 | 7.481 | 1.990 | 0.440 |
| Ha | Y | HD | 1 | 1.129 | 9.499 | 0.723 | -78.203 | 3.732 | 6.878 | 0.536 |
| Ha | Y | HD | 2 | 1.366 | 10.251 | 1.004 | -64.653 | 2.532 | 8.697 | 0.423 |
| Ha | Y | HD | 3 | 1.015 | 9.671 | 0.524 | -33.694 | 5.848 | 4.148 | 0.426 |
| Ha | Y | HU | 1 | 0.254 | 1.489 | 0.290 | 31.824 | 0.607 | 8.403 | 0.341 |
| Ha | Y | HU | 2 | 0.208 | 1.012 | 0.348 | -23.202 | 0.774 | 7.923 | 0.332 |
| Ha | Y | HU | 3 | 0.313 | 5.103 | 0.329 | -3.210 | 0.667 | 8.182 | 0.335 |
| Ha | Y | CV | 1 | 0.824 | 3.554 | 0.972 | -24.510 | 5.960 | 2.275 | 0.215 |
| Ha | Y | CV | 2 | 0.700 | 2.960 | 1.008 | -34.489 | 5.803 | 2.873 | 0.223 |
| Ha | Y | CV | 3 | 0.784 | 2.488 | 0.796 | -51.996 | 5.930 | 2.122 | 0.227 |
| Ha | Y | Mix | 1 | 1.053 | 9.917 | 0.714 | -43.169 | 6.995 | 1.523 | 0.324 |
| Ha | Y | Mix | 2 | 0.962 | 9.837 | 1.349 | -58.581 | 5.856 | 2.615 | 0.277 |
| Ha | Y | Mix | 3 | 0.881 | 9.018 | 1.078 | -47.269 | 6.558 | 1.720 | 0.338 |
| Ha | Ha | / | cntrl | 0.059 | 0.690 | 0.418 | -17.355 | 0.085 | 2.078 | 0.031 |
| Ha | Ha | / | cntrl | 0.091 | 0.498 | 0.542 | -8.946 | 0.023 | 2.036 | 0.065 |
| Ha | Ha | / | cntrl | 0.072 | 0.652 | 0.443 | -64.054 | 1.117 | 4.454 | 0.205 |
| Ha | Hu | / | cntrl | 0.059 | 0.690 | 0.418 | -17.355 | 0.085 | 2.078 | 0.031 |
| Ha | Hu | / | cntrl | 0.091 | 0.498 | 0.542 | -8.946 | 0.023 | 2.036 | 0.065 |
| Ha | Hu | / | cntrl | 0.072 | 0.652 | 0.443 | -64.054 | 1.117 | 4.454 | 0.205 |
| Ha | Y | / | cntrl | 0.059 | 0.690 | 0.418 | -17.355 | 0.085 | 2.078 | 0.031 |
| Ha | Y | / | cntrl | 0.091 | 0.498 | 0.542 | -8.946 | 0.023 | 2.036 | 0.065 |
| Ha | Y | / | cntrl | 0.072 | 0.652 | 0.443 | -64.054 | 1.117 | 4.454 | 0.205 |
| Hu | Ha | HD | 1 | 0.797 | 10.977 | 0.900 | -13.598 | 13.251 | 0.847 | 0.545 |
| Hu | Ha | HD | 2 | 0.073 | 1.324 | 0.933 | -50.119 | 13.222 | 1.048 | 0.395 |
| Hu | Ha | HD | 3 | 0.641 | 9.609 | 1.069 | -17.423 | 13.097 | 1.495 | 0.492 |
| Hu | Ha | HU | 1 | 0.190 | 1.682 | 0.398 | 0.838 | 4.778 | 6.372 | 0.493 |
| Hu | Ha | HU | 2 | 0.169 | 1.181 | 0.810 | -13.943 | 6.031 | 3.356 | 0.509 |
| Hu | Ha | HU | 3 | 0.134 | 1.478 | 0.581 | -28.530 | 5.797 | 3.745 | 0.492 |
| Hu | Ha | CV | 1 | 0.275 | 2.014 | 0.537 | -11.058 | 8.048 | 0.558 | 0.674 |
| Hu | Ha | CV | 2 | 0.591 | 2.867 | 0.646 | -16.046 | 6.637 | 11.459 | 1.031 |
| Hu | Ha | CV | 3 | 0.582 | 2.500 | 0.504 | -24.338 | 9.185 | 6.730 | 0.923 |
| Hu | Ha | Mix | 1 | 0.540 | 10.592 | 0.518 | -11.429 | 12.001 | 1.236 | 0.721 |
| Hu | Ha | Mix | 2 | 0.876 | 9.505 | 0.720 | -31.978 | 6.774 | 4.823 | 0.781 |
| Hu | Ha | Mix | 3 | 0.625 | 9.507 | 0.423 | -37.974 | 8.853 | 2.342 | 0.752 |
| Hu | Hu | HD | 1 | 1.719 | 10.709 | 1.752 | -7.928 | 14.848 | 0.643 | 0.483 |
| Hu | Hu | HD | 2 | 2.041 | 10.998 | 0.807 | -58.768 | 14.534 | 2.370 | 0.603 |
| Hu | Hu | HD | 3 | 1.212 | 9.835 | 1.318 | -61.465 | 13.341 | 2.718 | 0.625 |
| Hu | Hu | HU | 1 | 0.214 | 1.631 | 0.866 | -32.199 | 6.881 | 2.016 | 0.501 |
| Hu | Hu | HU | 2 | 0.193 | 1.044 | 0.297 | -44.373 | 5.770 | 3.981 | 0.470 |
| Hu | Hu | HU | 3 | 0.269 | 2.734 | 0.564 | -16.736 | 6.181 | 2.617 | 0.519 |
| Hu | Hu | CV | 1 | 0.240 | 3.382 | 0.595 | -37.403 | 7.939 | 6.651 | 0.970 |
| Hu | Hu | CV | 2 | 0.231 | 1.467 | 0.434 | -23.721 | 7.592 | 8.142 | 1.038 |
| Hu | Hu | CV | 3 | 0.290 | 4.041 | 1.068 | 4.033 | 7.296 | 9.822 | 1.021 |
| Hu | Hu | Mix | 1 | 1.441 | 10.092 | 0.416 | -55.431 | 13.182 | 1.439 | 0.622 |
| Hu | Hu | Mix | 2 | 0.837 | 9.332 | 0.921 | -52.307 | 12.040 | 3.058 | 0.760 |
| Hu | Hu | Mix | 3 | 1.046 | 9.221 | 0.491 | -60.298 | 12.240 | 3.152 | 0.876 |
| Hu | Y | HD | 1 | 0.903 | 10.218 | 0.491 | -36.421 | 13.287 | 2.354 | 0.493 |
| Hu | Y | HD | 2 | 1.247 | 10.675 | 0.592 | -80.054 | 12.922 | 1.761 | 0.475 |
| Hu | Y | HD | 3 | 1.294 | 9.483 | 0.758 | -82.670 | 6.013 | 4.183 | 0.592 |
| Hu | Y | HU | 1 | 0.194 | 2.373 | 0.598 | -136.942 | 6.315 | 3.237 | 0.486 |
| Hu | Y | HU | 2 | 0.227 | 1.498 | 0.478 | -38.950 | 5.939 | 3.699 | 0.496 |
| Hu | Y | HU | 3 | 0.181 | 2.680 | 0.722 | -82.200 | 7.629 | 5.211 | 0.790 |
| Hu | Y | CV | 1 | 0.438 | 2.952 | 0.460 | -66.684 | 4.948 | 12.557 | 0.867 |
| Hu | Y | CV | 2 | 0.745 | 3.159 | 0.379 | -119.992 | 8.386 | 5.595 | 0.821 |
| Hu | Y | CV | 3 | 0.612 | 2.123 | 0.347 | -65.441 | 9.277 | 4.533 | 0.764 |
| Hu | Y | Mix | 1 | 0.668 | 9.990 | 0.908 | -43.966 | 10.626 | 2.856 | 0.689 |
| Hu | Y | Mix | 2 | 0.748 | 7.347 | 0.876 | -78.148 | 7.913 | 2.657 | 0.766 |
| Hu | Y | Mix | 3 | 0.649 | 8.135 | 1.164 | -60.011 | 3.708 | 5.895 | 0.455 |
| Hu | Ha | / | cntrl | 0.106 | 1.023 | 0.429 | -48.926 | 4.384 | 4.890 | 0.437 |
| Hu | Ha | / | cntrl | 0.093 | 1.158 | 0.654 | -54.156 | 4.264 | 4.746 | 0.424 |
| Hu | Ha | / | cntrl | 0.047 | 1.158 | 0.541 | 14.475 | 1.762 | 9.999 | 0.459 |
| Hu | Hu | / | cntrl | 0.106 | 1.023 | 0.429 | -48.926 | 4.384 | 4.890 | 0.437 |
| Hu | Hu | / | cntrl | 0.093 | 1.158 | 0.654 | -54.156 | 4.264 | 4.746 | 0.424 |
| Hu | Hu | / | cntrl | 0.047 | 1.158 | 0.541 | 14.475 | 1.762 | 9.999 | 0.459 |
| Hu | Y | / | cntrl | 0.106 | 1.023 | 0.429 | -48.926 | 4.384 | 4.890 | 0.437 |
| Hu | Y | / | cntrl | 0.093 | 1.158 | 0.654 | -54.156 | 4.264 | 4.746 | 0.424 |
| Hu | Y | / | cntrl | 0.047 | 1.158 | 0.541 | 14.475 | 1.762 | 9.999 | 0.459 |
| Y | Ha | HD | 1 | 1.703 | 10.289 | 0.372 | -13.602 | 4.844 | 10.111 | 1.189 |
| Y | Ha | HD | 2 | 1.546 | 9.971 | 1.371 | -19.159 | 12.495 | 3.765 | 0.664 |
| Y | Ha | HD | 3 | 1.821 | 11.172 | 0.549 | -87.152 | 8.516 | 4.206 | 1.476 |
| Y | Ha | HU | 1 | 0.305 | 2.862 | 0.378 | -89.941 | 0.775 | 11.165 | 0.833 |
| Y | Ha | HU | 2 | 0.278 | 2.327 | 0.183 | -31.893 | 0.258 | 10.334 | 0.620 |
| Y | Ha | HU | 3 | 0.211 | 1.331 | 0.366 | -49.582 | 0.209 | 10.040 | 0.636 |
| Y | Ha | CV | 1 | 0.393 | 2.336 | 0.579 | -51.354 | 1.868 | 24.692 | 1.165 |
| Y | Ha | CV | 2 | 0.554 | 1.810 | 0.801 | -15.599 | 2.339 | 23.382 | 1.078 |
| Y | Ha | CV | 3 | 0.388 | 3.396 | 0.606 | -40.912 | 7.085 | 4.837 | 0.817 |
| Y | Ha | Mix | 1 | 1.052 | 10.244 | 0.970 | 4.442 | 3.421 | 12.617 | 1.242 |
| Y | Ha | Mix | 2 | 1.066 | 11.091 | 0.669 | -69.082 | 4.295 | 11.803 | 1.091 |
| Y | Ha | Mix | 3 | 1.231 | 10.651 | 1.347 | -40.063 | 3.178 | 12.664 | 1.436 |
| Y | Hu | HD | 1 | 2.178 | 10.017 | 0.678 | -68.074 | 6.039 | 5.571 | 1.451 |
| Y | Hu | HD | 2 | 2.189 | 10.898 | 1.055 | -115.622 | 7.793 | 2.671 | 2.087 |
| Y | Hu | HD | 3 | 2.672 | 10.789 | 1.385 | -103.869 | 7.374 | 5.659 | 2.267 |
| Y | Hu | HU | 1 | 0.344 | 1.162 | 0.398 | -43.434 | 0.238 | 10.509 | 0.638 |
| Y | Hu | HU | 2 | 0.332 | 1.770 | 0.419 | 15.983 | 0.380 | 9.473 | 0.602 |
| Y | Hu | HU | 3 | 0.370 | 1.703 | 0.397 | -8.297 | 0.268 | 9.920 | 0.597 |
| Y | Hu | CV | 1 | 0.473 | 1.925 | 1.063 | -68.062 | 4.015 | 17.760 | 1.642 |
| Y | Hu | CV | 2 | 0.573 | 3.057 | 0.411 | 24.636 | 4.447 | 16.875 | 1.501 |
| Y | Hu | CV | 3 | 0.632 | 2.042 | 1.195 | 42.424 | 1.696 | 25.611 | 1.869 |
| Y | Hu | Mix | 1 | 1.918 | 11.131 | 0.378 | -46.170 | 4.806 | 12.139 | 1.573 |
| Y | Hu | Mix | 2 | 2.924 | 10.777 | 0.719 | 14.968 | 4.194 | 12.372 | 1.586 |
| Y | Hu | Mix | 3 | 2.364 | 10.593 | 0.921 | -83.518 | 3.576 | 12.546 | 1.517 |
| Y | Y | HD | 1 | 2.296 | 10.995 | 1.041 | -43.345 | 7.181 | 3.872 | 1.518 |
| Y | Y | HD | 2 | 1.602 | 10.470 | 1.172 | -86.256 | 5.794 | 5.828 | 1.409 |
| Y | Y | HD | 3 | 2.101 | 11.045 | 0.931 | -117.625 | 4.961 | 8.316 | 1.713 |
| Y | Y | HU | 1 | 0.271 | 1.386 | 0.443 | -40.121 | 0.493 | 8.039 | 0.545 |
| Y | Y | HU | 2 | 0.298 | 3.655 | 0.304 | -58.421 | 0.538 | 10.768 | 0.685 |
| Y | Y | HU | 3 | 0.263 | 1.085 | 0.391 | 58.650 | 0.552 | 11.138 | 0.696 |
| Y | Y | CV | 1 | 0.357 | 4.600 | 0.498 | 0.439 | 2.177 | 19.776 | 1.368 |
| Y | Y | CV | 2 | 0.360 | 2.786 | 0.253 | 5.696 | 2.676 | 12.571 | 1.065 |
| Y | Y | CV | 3 | 0.341 | 2.622 | 0.807 | -50.479 | 2.349 | 13.325 | 1.326 |
| Y | Y | Mix | 1 | 1.767 | 10.024 | 0.818 | -67.061 | 3.966 | 10.666 | 1.088 |
| Y | Y | Mix | 2 | 1.326 | 10.343 | 0.378 | -61.574 | 6.171 | 5.079 | 0.878 |
| Y | Y | Mix | 3 | 1.327 | 10.758 | 0.378 | -39.049 | 3.288 | 12.727 | 1.445 |
| Y | Ha | / | cntrl | 0.258 | 1.190 | 0.405 | -20.218 | 0.217 | 9.758 | 0.600 |
| Y | Ha | / | cntrl | 0.305 | 2.561 | 0.967 | -30.673 | 0.223 | 8.622 | 0.548 |
| Y | Ha | / | cntrl | 0.346 | 1.289 | 0.461 | -89.555 | 0.424 | 9.865 | 0.582 |
| Y | Hu | / | cntrl | 0.258 | 1.190 | 0.405 | -20.218 | 0.217 | 9.758 | 0.600 |
| Y | Hu | / | cntrl | 0.305 | 2.561 | 0.967 | -30.673 | 0.223 | 8.622 | 0.548 |
| Y | Hu | / | cntrl | 0.346 | 1.289 | 0.461 | -89.555 | 0.424 | 9.865 | 0.582 |
| Y | Y | / | cntrl | 0.258 | 1.190 | 0.405 | -20.218 | 0.217 | 9.758 | 0.600 |
| Y | Y | / | cntrl | 0.305 | 2.561 | 0.967 | -30.673 | 0.223 | 8.622 | 0.548 |
| Y | Y | / | cntrl | 0.346 | 1.289 | 0.461 | -89.555 | 0.424 | 9.865 | 0.582 |

**
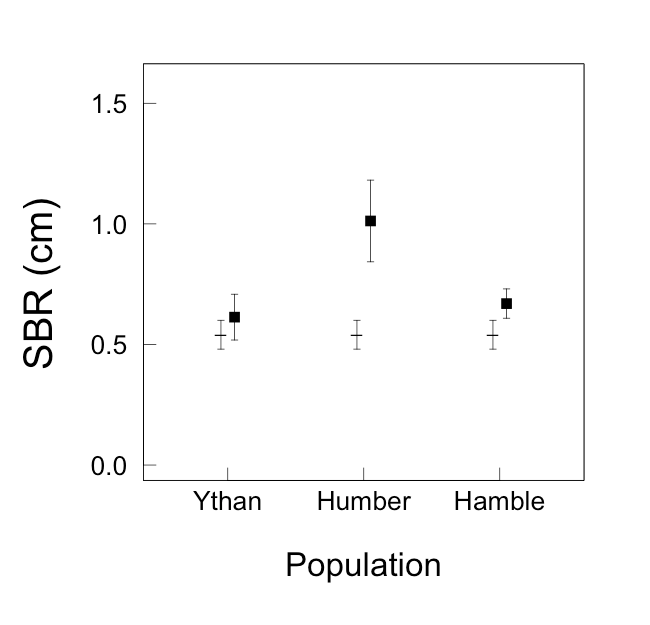
**

Figure S2: The effects of population on surface boundary roughness (SBR, mean ± s.e., n = 3) for *Corophium volutator* (squares). Observations without macrofauna (dash, n = 9) are shown for comparison.


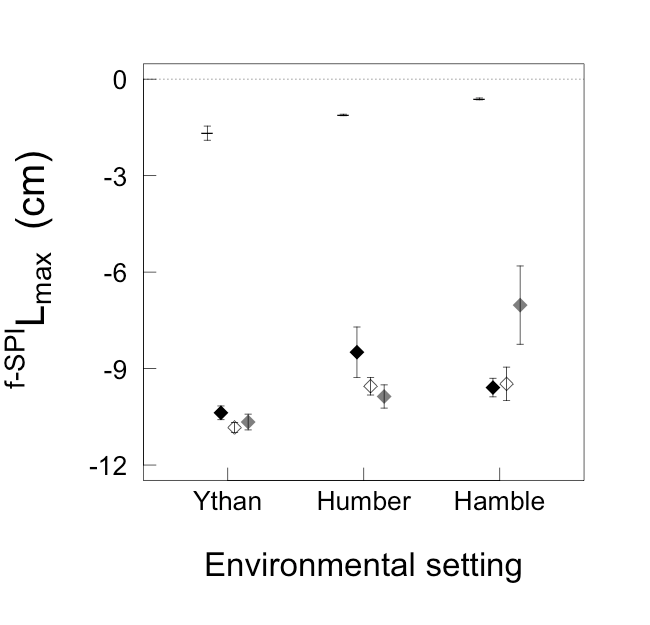


Figure S3: The interactive effect of environmental setting and population on the maximum depth of sediment particle reworking (^f-SPI^L_max_, cm, mean ± s.e., n = 3) for the species mixture (diamonds). Observations without macrofauna (dash, n = 9) are shown for comparison. Shadings indicate different populations: black = Ythan Estuary, white = Humber Estuary, grey = Hamble Estuary. The dotted line indicates the sediment surface and negative values indicate deeper net downward transport of sediment particles.

**
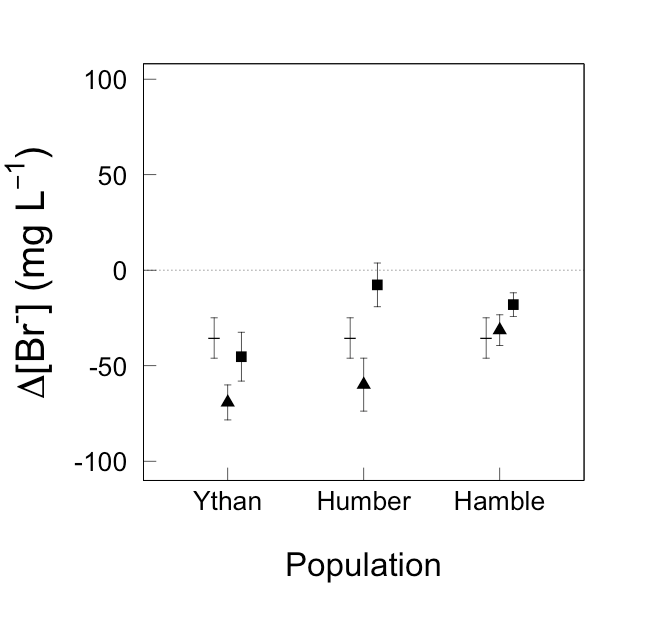
**

Figure S4: The effect of population on burrow ventilation activity (Δ[BR^-^], mg L^-1^, mean ± s.e., n = 3) for *Hediste diversicolor* (triangles) and *Corophium volutator* (squares). Observations without macrofauna (dash, n = 9) are shown for comparison. Negative values indicate increased activity.

**
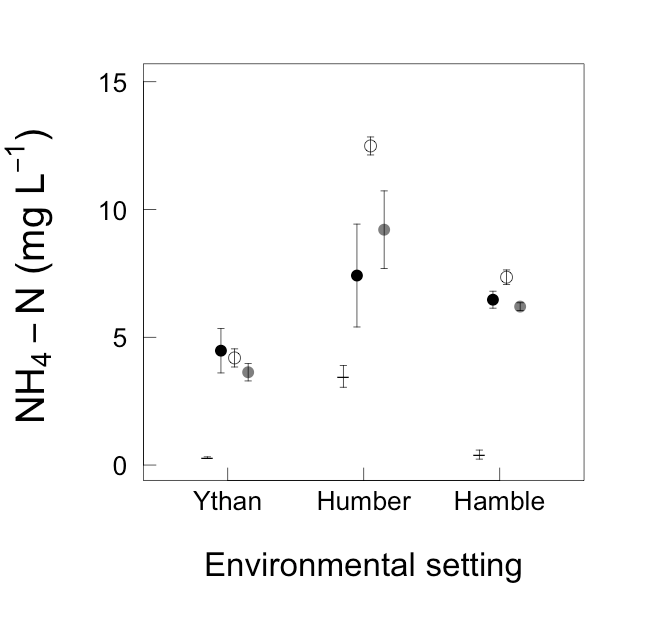
**

Figure S5: The interactive effect of environmental setting and population on [NH_4_-N] (mg L^-1^, mean ± s.e., n = 3) for *Hydrobia ulvae* (circles)*.* Observations without macrofauna (dash, n = 9) are shown for comparison. Shadings indicate different populations: black = Ythan Estuary, white = Humber Estuary, grey = Hamble Estuary.

**References**

Blott, S. J. & Pye, K. Technical Communication Gradistat: a Grain Size Distribution and Statistics Package for the Analysis of Unconsolidated Sediments. *Earth Surface Processes and Landforms* **26,** 1237–1248 (2001).
